# Fabrication of Loose Nanofiltration Membrane by Crosslinking TEMPO-Oxidized Cellulose Nanofibers for Effective Dye/Salt Separation

**DOI:** 10.3390/molecules29102246

**Published:** 2024-05-10

**Authors:** Shasha Liu, Mei Sun, Can Wu, Kaixuan Zhu, Ying Hu, Meng Shan, Meng Wang, Kai Wu, Jingyi Wu, Zongli Xie, Hai Tang

**Affiliations:** 1 School of Chemical and Environmental Engineering, Anhui Polytechnic University, Wuhu 241000, China; liushasha@ahpu.edu.cn (S.L.); 2230621105@stu.ahpu.edu.cn (M.S.); wucancz@163.com (C.W.); kaixuan0080@163.com (K.Z.); huying@ahpu.edu.cn (Y.H.); 2220630110@stu.ahpu.edu.cn (M.S.); wm18855372043@163.com (M.W.); 13955064236@163.com (K.W.); wujingyi0602@163.com (J.W.); 2 CSIRO Manufacturing, Private Bag 10, Clayton South, VIC 3169, Australia

**Keywords:** TEMPO-oxidized cellulose nanofibers, nanofiltration membrane, dye/salt separation

## Abstract

Dye/salt separation has gained increasing attention in recent years, prompting the quest to find cost-effective and environmentally friendly raw materials for synthesizing high performance nanofiltration (NF) membrane for effective dye/salt separation. Herein, a high-performance loose-structured NF membrane was fabricated via a simple vacuum filtration method using a green nanomaterial, 2,2,6,6-tetramethylpiperidine-1-oxide radical (TEMPO)-oxidized cellulose nanofiber (TOCNF), by sequentially filtrating larger-sized and finer-sized TOCNFs on a microporous substrate, followed by crosslinking with trimesoyl chloride. The resulting TCM membrane possessed a separating layer composed entirely of pure TOCNF, eliminating the need for other polymer or nanomaterial additives. TCM membranes exhibit high performance and effective dye/salt selectivity. Scanning Electron Microscope (SEM) analysis shows that the TCM membrane with the Fine-TOCNF layer has a tight layered structure. Further characterizations via Fourier transform infrared spectroscopy (FTIR) and X-ray diffraction (XRD) confirmed the presence of functional groups and chemical bonds of the crosslinked membrane. Notably, the optimized TCM-5 membrane exhibits a rejection rate of over 99% for various dyes (Congo red and orange yellow) and 14.2% for NaCl, showcasing a potential candidate for efficient dye wastewater treatment.

## 1. Introduction

The rapid development of the global textile industry has heightened the urgency for treating dye wastewater and addressing environmental pollution [1]. Traditional methods for treating dye wastewater often face challenges such as low efficiency, high cost, and the generation of large amounts of by-products [2,3]. In the textile industry, the synthesis or application of dyes typically leads to the generation of high-salinity-dye wastewater [4]. The dye synthesis process yields a large amount of inorganic salt (i.e., ~5.0% NaCl) as a by-product, diminishing the dye’s purity and reducing the brightness of the printed image in textile applications [5,6,7]. Discharging a large amount of salt along with dyes is the main issue in textile wastewater [8,9,10]. Therefore, effective treatment of textile wastewater is of great significance to mitigate the release of the highly polluted dye wastewater. Various approaches, including biotechnology and adsorption, have been applied to treat dye wastewater. However, most of these solutions face problems such as toxic nanomaterials leaching, limited flexibility, high cost, and complicated operating processes. Therefore, the identification of green materials and simple procedures is crucial to solving this problem.

Cellulose is a polysaccharide with a crystalline structure that is usually derived from abundant and renewable plants. As a member of the cellulose nanomaterial family, 2,2,6,6-tetramethylpiperidine-1-oxide radical (TEMPO)-oxidized cellulose nanofiber (TOCNF) is synthesized in a mild aqueous environment, followed by moderate mechanical treatment. Compared with other cellulose nanomaterials fabrication approaches, such as high-pressure homogenization and acid hydrolysis, the TEMPO-mediated oxidation method is more environmentally friendly. TOCNF has many advantages, such as a high surface-to-volume ratio, excellent mechanical stability, multi-functional surface groups, cost-effectiveness, and environmentally friendly characteristics [11]. TOCNF has made significant breakthroughs in the fields of adsorption and separation [12,13,14,15]. Through processing and functional modification of TOCNF, it can be applied to the preparation of nanofiltration (NF) or reverse osmosis (RO) membranes for dye wastewater treatment [16]. Due to its hydrogen-bonded parallel chains, TOCNF is a strong natural nanomaterial with a one-dimensional (1D) structure [17,18]. It can be combined with other two-dimensional (2D) nanomaterials to fabricate NF membranes [19,20]. Yang et al. [19] reported a mixed-dimensional NF membrane fabricated by assembling TOCNFs and covalent organic framework (COF) nanosheets. The prepared TOCNF/COF NF membrane exhibited outstanding hydrolytic stability and improved mechanical properties. Mohammed et al. [20] developed an NF membrane by incorporating graphene oxide (GO) nanosheets into the CNF matrix via the vacuum filtration method, followed by crosslinking by glutaraldehyde. The obtained GO/CNF mixed-dimensional NF membrane showed a pure solvent flux of 13.9 L m^−2^ h^−1^ bar^−1^ for water and over 90% rejections for two dyes.

Additionally, TOCNFs can serve as additives or substrates for thin composite NF/RO membrane [21,22,23]. Wang et al. [23] prepared a composite RO membrane with modified TOCNFs incorporated in a polyamide barrier layer based on an electrospun nanofibrous substrate. The inclusion of TOCNF improved both the flux and the rejection of the composite RO membrane and was attributed to the formation of external water channels caused by TOCNFs. In our previous work, TOCNFs were used as additives that were incorporated into the polyamide selective layer of the RO membrane to enhance the water flux and hydrophilicity of the membrane [22]. However, few researchers have focused on using pure TOCNF to fabricate a dense separating layer of an NF membrane.

Herein, we develop an environmentally friendly NF membrane with a hierarchical nanostructured TOCNF separating layer via a simple vacuum filtration method. Different diameters of TOCNFs were vacuum filtrated on polyvinylidene fluoride (PVDF) substrate membrane to form a dense NF membrane with a narrow pore size, which can effectively reject dyes. Specifically, Thick-TOCNFs (without probe ultrasonication) were vacuum-filtrated on the substrate membrane, followed by the addition of Fine-TOCNFs (with probe ultrasonication) to form a compact barrier layer of NF membrane. The Fine-TOCNFs served to further reduce the pore size of the composite membrane, consequently enhancing dye rejection rate. Furthermore, TMCs were used as a crosslinking agent to enhance the stability of the TOCNF layer. The morphology and physicochemical properties of the fabricated TOCNF NF membrane with different Fine-TOCNF concentrations were characterized, and their dye/salt separation performance was also evaluated compared with that of the neat Thick-TOCNF membrane. For better comparison, Fine-TOCNF membranes crosslinked with different concentrations of TMC were also synthesized. To the best of our knowledge, this work is the first study to use pure TOCNFs to fabricate the separating layer of NF membrane.

## 2. Result and Discussion

### 2.1. Morphology of TOCNFs and TCM Membranes

**Figure 1:**
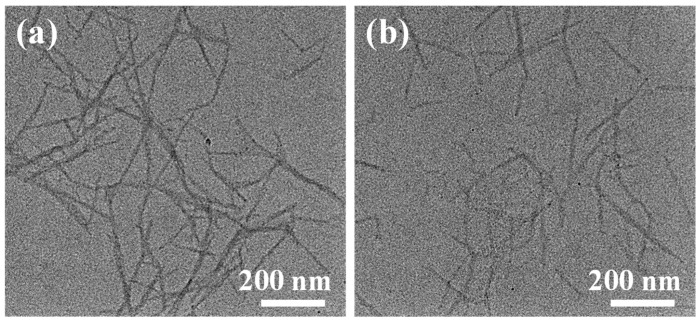
TEM images of (**a**) Thick-TOCNFs and (**b**) Fine-TOCNFs.

The morphologies of the Fine-TOCNFs and Thick-TOCNFs were observed by Transmission Electron Microscopy (TEM) (Figure 1). The length and diameter distributions of Fine-TOCNFs and Thick-TOCNFs are shown in Figure 1. The results show that the Thick-TOCNFs exhibit a long rod structure, while the Fine-TOCNFs show a shorter and finer rod structure. The length of Thick-TOCNFs is in the range of 300–1100 nm, and the average length is about 802 nm. The diameter of Thick-TOCNFs is 20.8 nm. After the probe ultrasonic treatment, the Fine-TOCNFs became shorter and thinner, the average length of Fine-TOCNFs reduced to 476 nm, and average width of Fine-TOCNFs decreased to 14.8 nm. The results show that compared to Thick-TOCNFs, Fine-TOCNFs became shorter and thinner, mainly due to the powerful probe ultrasonication, and thus the cellulose dispersed more evenly. In this study, Thick-TOCNFs without probe ultrasound were longer and were used to fill the big holes in the PVDF substrate membrane (pore size 220 nm). Then, Thin-TOCNFs were filtrated and crosslinked on the prefabricated membrane to form a denser layer, due to their finer diameter.

### 2.2. Characterization of Membranes

**Figure 2:**
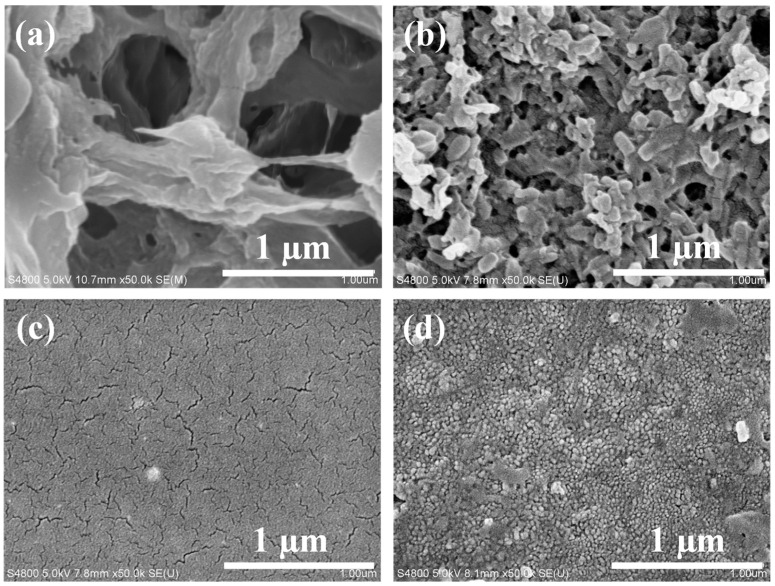
SEM images of membrane surface: (**a**) PVDF membrane, (**b**) TCM-0 membrane, (**c**) TCM-5 membrane, and (**d**) TCM-10 membrane.

Figure 2 shows the Scanning Electron Microscope (SEM) surface morphology images of the PVDF substrate membrane, TCM-0 membrane (5 mL Thick-TOCNFs + 0 mL Fine-TOCNFs), TCM-5 membrane (5 mL Thick-TOCNFs + 5 mL Fine-TOCNFs), and TCM-10 membrane (5 mL Thick-TOCNFs + 10 mL Fine-TOCNFs). The average pore size of PVDF membrane is ~220 μm. Some huge pores (500~600 μm) are observed in Figure 2a, while the pore size of TCM-0 membrane (Figure 2b) became much smaller (the average pore size is ~30 nm). TCM-0 membrane was prepared by vacuum filtrating 5 mL on PVDF membrane. The Thick-TOCNFs were uniformly distributed on the surface of the PVDF membrane, with obviously reduced surface pore size. However, this pore size was still not small enough to reject the dye molecules. Therefore, Thick-TOCNFs have a great effect on narrowing the substrate pore size. Based on this Thick-TOCNFs-modified substrate, an additional 5 mL or 10 mL of Fine-TOCNFs was filtrated and followed by TMC crosslinking, by which the TCM-5 and TCM-10 membrane were obtained. Both TCM-5 and TCM-10 membranes exhibited a uniform and compact surface structure, which can provide a possibility to reject dyes. No obvious big pores could be observed in the TCM-5 and TCM-10 membranes under 50 k magnification. The pore sizes of TCM-5 and TCM-10 were about several nanometers.

**Figure 3:**
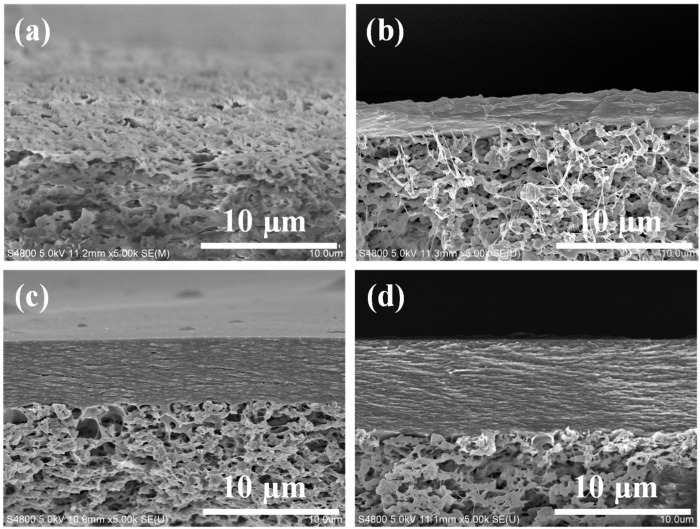
SEM images of membrane cross-sections: (**a**) PVDF membrane, (**b**) TCM-0 membrane, (**c**) TCM-5 membrane, and (**d**) TCM-10 membrane.

Figure 3 shows a cross-sectional SEM image of the TCM membranes. Compared with the substrate membrane, TCM-0 membrane has an extra top layer (2.53 μm). As the content of Fine-TOCNFs increases, the thickness of the extra top layer of the composite membrane also increases. The top layer thicknesses of TCM-5 and TCM-10 are about 4.75 nm and 6.66 nm, respectively. This result indicates that more and more cellulose fibers assemble in the top layer with the increase in the Fine-TOCNFs concentration. Moreover, the image reveals that the top layers of TCM-5 and TCM-10 membranes are more compact, presenting a tight layered stacking structure. This is mainly due to the Fine-TOCNFs filling the pores of the Thick-TOCNFs layer, creating a denser separation layer. The compact top layer is beneficial for the high rejection rate of the membranes. The separation performance of TCM-0, TCM-5, and TCM-10 membranes shows that TCM-10 membrane possesses the highest rejection rate of Congo red (CR) dye.

**Figure 4:**
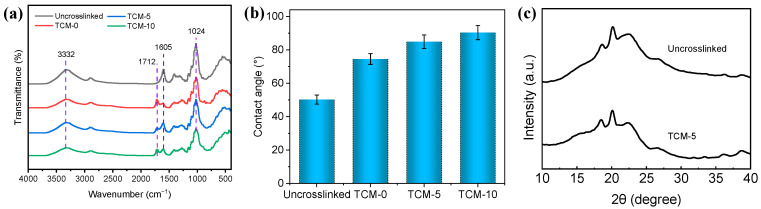
(**a**) FTIR, and (**b**) CA of Uncrosslinked, TCM-0, TCM-5, and TCM-10 membrane; (**c**) XRD of Uncrosslinked membrane and TCM-5 membrane.

Figure 4a shows the Fourier transform infrared spectroscopy (FTIR) spectra of membrane samples, including the control (TOCNFs content equivalent to TCM-5 but without TMC crosslinking process), TCM-0 membrane, TCM-5 membrane, and TCM-10 membrane sample. The characteristic vibrational bands of TOCNFs are typically observed near 3322 cm^−1^ and 1024 cm^−1^, corresponding to the hydroxyl and cyclic alcohol groups in CNFs, respectively [22]. Additionally, the vibrational band at 1605 cm^−1^ is the C=C ring stretching vibration. Compared to the Uncrosslinked membrane sample, all TOCNF membranes crosslinked with TMC (TCM-0, TCM-5, and TCM-10 membranes) exhibit an additional vibrational band at 1712 cm^−1^, indicating the presence of the ester bond generated via the acylation reaction between the acyl chloride groups of the TMC and -OH groups of TOCNF [24]. This vibrational band confirms the successful crosslinking between TMC and TOCNFs on the membrane’s surface. Furthermore, in comparison to the Uncrosslinked membrane without TMC crosslinking, the crosslinked membranes (TCM-0, TCM-5, and TCM-10 membranes) show lower transmittance vibrational bands near 3322 cm^−1^ and 1023 cm^−1^, with the intensity of these two vibrational bands decreasing as the concentration of Fine-TOCNFs increases. This result suggests that the increase in -OH group concentration due to TOCNFs may increase the membrane hydrophilicity, while crosslinking agent TMC could weaken the membrane hydrophilicity.

Figure 4b shows the water contact angle of Uncrosslinked, TCM-0, TCM-5, and TCM-10 membrane samples. It can be seen that increasing the Fine-TOCNF content gradually weakens the hydrophilicity, which may be related to the increased hydrophobicity of TOCNF after TMC crosslinking. With the increase in TOCNF content, the crosslinked TOCNFs also increased, causing a slight increase in the contact angle, which was consistent with the infrared spectroscopy results.

The X-ray diffraction (XRD) patterns of TOCNF membrane before and after TMC crosslinking are shown in Figure 4c. The result shows similar peaks at 2θ = 18° and 20° for both the TOCNF membrane, with and without TMC crosslinking. However, the TOCNF membrane after TMC crosslinking shows a weaker peak at 2θ = 22.5°, corresponding to the (002) crystal plane of cellulose I [25]. This small difference may show that TMC crosslinking weakened the crystalline structure of TOCNF membrane.

### 2.3. The Performance of TCM Membranes

**Figure 5:**
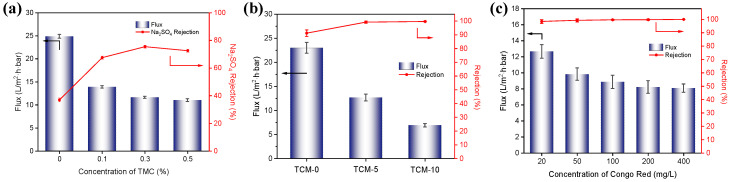
(**a**) The pure water flux and rejection of Na_2_SO_4_ solution (1000 ppm) of TCM membrane crosslinked with different TMC concentration. (**b**) The pure water flux and rejection of CR dye by TCM membranes with different Fine-TOCNF contents (TCM-0, TCM-5, TCM-10 membrane). (**c**) The flux and rejection of CR dye by the TCM-5 membrane at different concentrations.

The effect of crosslinking agent (TMC) concentration on the pure water flux and Na_2_SO_4_ rejection performance of the composite membranes was investigated (Figure 5a). All of the membrane samples were prepared with the same method as the TCM-5 membrane sample, except for the crosslinking agent (TMC) concentration. Compared with the Uncrosslinked membrane, the membrane crosslinked with 0.1% TMC showed a decrease in pure water flux of the nanofiltration membrane from 24.87 L/m^2^·h·bar to 13.89 L/m^2^·h·bar, and the Na_2_SO_4_ rejection rate increased from 36.9% to 67.6%. This indicates an improvement in the membrane’s rejection performance after TMC crosslinking, but a decrease in the water flux. This is mainly due to the consumption of hydrophilic carboxyl groups on the surface of the TMC crosslinked membrane, resulting in decreased hydrophilicity and reducing the rate of water molecule transport through the membrane, thereby decreasing the flux. With a further increase in TMC concentration, the flux slightly decreased, but the Na_2_SO_4_ rejection effect first increased and then decreased. This is mainly because the increase in TMC concentration helps to improve the crosslinking degree of TOCNFs and provides a denser surface, thus enhancing the membrane’s rejection performance. However, a high crosslinking degree reduces the water flux, while the pore size sieving effect cannot further improve the Na_2_SO_4_ rejection rate. When the TOCNFs were not crosslinked with TMC, the structure formed by the hydrogen bonding between the TOCNFs was relatively loose and not strong. Therefore, when the TMC concentration was 0.3%, the nanofiltration membrane had an optimal rejection performance, with a Na_2_SO_4_ rejection rate of 75.5% and a pure water flux of 11.61 L/m^2^·h·bar.

The effect of the Fine-TOCNF content on the separation performance of TCM membranes can be seen in Figure 5b, where the flux decreases as the TOCNF content increases. As the SEM images (Figure 3) and contact anger test (Figure 4b) results show, with the increase in Fine-TOCNF content, the thickness of the top layer increases, and the hydrophilicity of the membranes decreases. Both the thicker selective layer and decreased hydrophilicity of the membrane may lead to more resistance for water molecules attempting to pass through the membrane. On the other hand, the rejection rate of CR increased rapidly after adding Fine-TOCNFs, and the CR rejection rate of TCM-5 membrane increased from 91.2% to 99.2% compared to TCM-0, and further increased to 99.7% for the TCM-10 membrane. This indicates that the dense pore structure produced by probe-ultrasound-treated TOCNF (Fine-TOCNF) has an excellent rejection effect on small-molecule dyes. The results show that by increasing the CNF content loaded on the membrane surface, the rejection rate of the membrane can be improved to a certain extent, but the flux decreases to a certain extent. TCM-5 membrane still has a rejection rate of 99.2% for CR while retaining a certain permeability performance (12.67 L/m^2^·h·bar).

The impact of different concentrations of CR on the performance of nanofiltration membranes was tested by adjusting the dye concentration from 20 to 400 ppm (Figure 5c). The flux of the TCM-5 membrane slightly decreased with the increase in the dye concentration, from 12.66 L/m^2^·h·bar to around 8.09 L/m^2^·h·bar. This is because as the dye concentration increases, the dye molecules are more likely to attach to the pores and permeation channels on the membrane surface, forming a dense layer of dye molecules which increases the transmembrane resistance of water molecules and leads to a decrease in water flux. In addition, as the dye concentration increases, the membrane’s rejection rate of dye also increases slightly from 98.4% to 99.9%. This is mainly due to the formation of a “filter cake” layer on the membrane surface, which creates a spatial resistance effect that blocks the passage of dye molecules, resulting in a decrease in water flux but an increase in the membrane’s rejection rate of dye.

### 2.4. Separation Performance of TCM Membrane for Different Salts and Dyes

**Figure 6:**
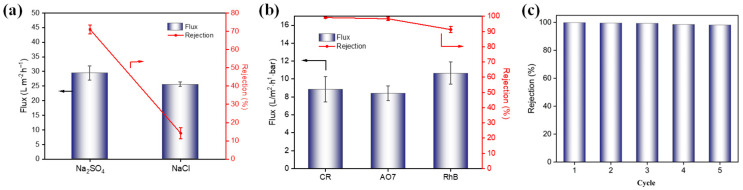
Separation performance of TCM-5 membrane for (**a**) Na_2_SO_4_ and NaCl, and (**b**) different dyes, including CR, AO7, and RhB. (**c**) Reusability test results of TCM-5 membrane against CR (200 ppm).

The salt separation performance of the TCM-5 membrane was evaluated based on the rejection of Na_2_SO_4_ and NaCl salt solutions at a pressure of 0.4 MPa, and the results of the permeation flux and rejection ratios are shown in Figure 6a. The TCM-5 membrane shows a good rejection performance for divalent salt, with rejection of 71.0% for Na_2_SO_4;_ and poor rejection for monovalent salt, with rejection of 14.2% for NaCl. The result is consistent with literature reports on negatively charged nanofiltration membrane performance [26]. According to the Donnan effect theory, the negatively charged nanofiltration membrane has a stronger repulsion force for divalent anions (SO_4_^2−^) than monovalent anions (Cl^−^), resulting in a higher rejection rate of Na_2_SO_4_ by the membrane. This result shows that TCM-5 membrane has good NaCl/Na_2_SO_4_ selectivity. Similarly, the low rejection rate of Rhodamine B (RhB) dyes with positive charges on the negatively charged membrane surface indicates that the separation mechanism of the composite membrane is a combination of pore size screening and electrostatic effects.

The dye selectivity performance of the TCM-5 membrane was evaluated by filtration of three types of dye solutions (CR, orange yellow (AO7), and RhB), and the results are shown in Figure 6b. The concentration of the dye was 200 mg/L, and the flux and dye of the membrane were tested at room temperature under 0.4 MPa using cross-flow equipment. Both CR and AO7 are cationic dyes, while RhB dye is an anodic dye. The rejection rate of TMC-5 membrane reached over 99% for both CR and AO7, while the rejection rate for RhB was only 91%. Generally, the removal rate of the membrane for these three dyes is mainly determined by their charge performance and molecular weight. Similar to the salt selectivity result shown in Figure 6a, the negatively charged NF membrane has a stronger repulsion force for cationic dyes than anodic dyes. Therefore, The TCM-5 membrane shows excellent rejection rates for CR and AO7. In terms of cationic dyes, the membrane will show higher rejection rates for dyes with larger molecular weight. Hence, the rejection rate of CR (Mw = 670 g/mol) is greater than that of AO7 (Mw = 350 g/mol). However, for RhB (Mw = 479 g/mol), the rejection rate is lower than that of AO7 with a larger molecular weight. This is mainly because the nanofiltration membrane surface is negatively charged, resulting in greater electrostatic repulsion of AO7 dye than RhB, and the combined effect of pore size and electrostatic effects leads to a lower rejection rate for RhB dye. The experimental results show that the prepared TCM-5 membrane has an excellent rejection effect for cationic dyes.

The reusability of the membrane was tested using cyclic filtration in cross-flow equipment (Figure 6c). Between each cycle, ethanol and distilled water were sequentially filtrated to wash the membrane. This washing method can remove the dyes from the membrane, due to the weaker electrostatic interactions between the dye and membrane [27]. After five cycles, the membrane still maintained a high rejection rate of 98.1%. The high rejection rate of TCM-5 membrane towards CR dye was mainly determined by pore size sieving and electrostatic interaction.

## 3. Experimental Section

### 3.1. Materials

1,3,5-benzenetriacyl chloride (TMC), 2,2,6,6-tetramethylpiperidine-1-oxide radical (TEMPO), sodium hypochlorite (NaClO), sodium bromide (NaBr), Congo red (CR), rhodamine B (RhB), orange yellow (AO7), Na_2_SO_4_, MgSO_4_, NaCl, and MgCl_2_ were purchased from Shanghai Aladdin Biochemical Co. Ltd. (Shanghai, China). Polyvinylidene fluoride (PVDF) membranes were purchased from Nantong Longjin Film Technology Co., Ltd. (Nantong, China). Kraft pulp (Kinleith Hi White) was provided by Oji Fibre Solutions Company (Jinan, China). All the chemicals were used as received without further purification. Deionized (DI) water was used to prepare all aqueous solutions.

### 3.2. Preparation of TOCNF

A TOCNFs suspension was synthesized using a similar method to one that was recently reported in the literature [22]. To be specific, 1g pulp was used as raw material, shredded and dissolved in 100 mL of DI water, and added to 100 mg of NaBr, 16 mg of TEMPO, and 11.16 g of NaClO (the original concentration was 12.5%). The pH of the mixed solution was adjusted to 10 and maintained for 8 h. Then, centrifugation was performed three times at 9000 rpm to remove any remaining impurities from the mixture to obtain a gel-like solid. Then, 500 mL of DI water was added to dissolve the solid, and the mixture was stirred in a homogenizer for 3 min to obtain a transparent aqueous solution. The obtained solution was named Thick-TOCNFs. Finally, some of the obtained Thick-TOCNF solution was further broken by an ultrasonic probe, using a Qsonica sonicator with a pulse of 30% and output power of 500 W-20 kHz to yield a transparent solution. The obtained transparent TOCNF solution was named Fine-TOCNF. The concentration of both the Thick-TOCNF and Fine-TOCNF was 0.2 wt%.

### 3.3. Preparation of TCM

A vacuum-assistant method was adopted to prepare the TOCNF membranes. The specific steps were as follows: Firstly, 5 mL Thick-TOCNF solution was filtrated onto a PVDF membrane (pore size 200 nm). Then, an additional 0, 5, or 10 mL Fine-TOCNF solution was filtered onto the membrane, respectively. After the TOCNF solutions were vacuumed, 5 mL TMC solution (0.3% in n-hexane) was poured on the membrane to crosslink the TOCNFs. Finally, the obtained TOCNF membranes were kept in an oven at 80 °C for 30 min for better crosslinking. Based on the different Fine-TOCNF dosage, the obtained membranes were named TCM-0, TCM-5, and TCM-10, respectively. As a comparison, a control membrane (Uncrosslinked membrane) was prepared in the same condition as TCM-5 but without the TMC crosslinking step. All the prepared membranes were rinsed and stored in DI water.

### 3.4. Characterization of Membranes

The morphologies of Thick-TOCNFs and Fine-TOCNFs were observed by TEM (FEI Tecnai F20, Hillsboro, OR, USA). Diluted Thick-TOCNFs and Fine-TOCNFs drops were dropped on copper grids. The surface and cross-section morphologies of the PVDF membrane, TCM-0, TCM-5, and TCM-10 membrane were observed by SEM. All the samples were coated with gold, and the cross-section membrane samples were fractured in liquid nitrogen to maintain the membranes’ original morphology. The thickness of the membrane’s top layer was measured by Nano Measurer 1.2.5 software, and the thickness value was the average value of at least ten measurements. The chemical compositions of Uncrosslinked membrane, TCM-0, TCM-5, and TCM-10 membranes were obtained through FTIR (Thermo Scientific Nicolet iS 10, Waltham, MA, USA) in the wavelength range of 400–4000 cm^−1^. The hydrophilicity of PVDF membrane, TCM-0, TCM-5, and TCM-10 membranes was measured at room temperature using a contact-angle measuring instrument (OSA 60, Königshofen, Germany). The contact angle was tested based on a video camera image of the droplet using the drop shape analysis software (SurfaceMeter Element) supplied. The contact angles of water drops were measured in quintuplicate to obtain the average contact angle. The crystal structure of cellulose in Uncrosslinked membrane and TCM-5 membranes were measured by a XRD (Rigaku Miniflex 600, Yamanashi, Japan). XRD patterns were determined at 40 kV and 25 mA.

### 3.5. Permeability Performance of TCM Membrane

The filtration performance of composite membranes was evaluated using a cross-flow filtration system. Membrane flux and rejection rate tests were conducted on an effective membrane surface area of 25.12 cm^2^ at 0.4 MPa. Dye or salt solutions were used as feed solution, and the weight of the permeate solution was continuously recorded using a precision electronic balance connected to a computer. All results given are averages with standard deviations for at least three samples of each type of membrane. The pure water flow rate was calculated using Equation (1). The dye or salt rejection rate was calculated using Equation (2).

(1) $$F=\frac{V}{A\times \Delta t},$$

where *F* is the permeate flux (L/m^2^h), *V* is the permeate volume (L), *A* is the membrane area (m^2^), and Δ*t* is the filtration time (h).

(2) $$R=\left(1-\frac{C_{P}}{C_{f}}\right)\times 100\%,$$

where *R* is the rejection, and *C_p_* and *C_f_* are the dye or salt concentrations of permeate and feed solution, respectively.

The dye rejection test was carried out on TCM-5 membrane using cross-flow equipment at under 0.4 MPa, and three different dyes (CR, AO7, and RhB) were used for the dye rejection test using dye solution (200 ppm). The concentration of dyes was determined by a UV spectrophotometer. For the salt rejection test, 1000 ppm Na_2_SO_4_ or NaCl solution was used as feed solution, and the salt concentration of the permeate and feed solution was measured by an electrical conductivity device.

## 4. Conclusions

In this work, TCM nanofiltration membranes were fabricated by depositing Fine-TOCNF into Thick-TOCNF structure, followed by crosslinking with TMC. The dense selective layer of the TCM nanofiltration membrane consisted entirely of pure cellulose nanofibers. The fabrication conditions were optimized, and the results show that 0.3% TMC as the crosslinking agent was the optimal concentration, and Fine-TOCNF 5 mL filtrated on the Thick-TOCNF membrane was the optimal fabrication condition. The SEM result shows that TCM-5 and TCM-10 membranes have a tighter pore structure compared with the TCM-0 membrane (without Fine-TOCNF) layer. The hydrophilicity of the TCM membranes decreased as the Fine-TOCNF content increased. The TCM-5 membrane shows a good rejection rate for divalent salt (Na_2_SO_4_) with a rejection rate of 71.0% and a low rejection of monovalent ion (NaCl) at only 14.2%. Meanwhile, the TCM-5 membrane shows excellent removal performance (over 90%) for all three of the tested dyes, especially for CR and AO7 (both of which showed removal of over 99.0%). These findings highlight the TCM-5 membrane’s exceptional dyes/NaCl selectivity and NaCl/Na_2_SO_4_ selectivity. Moreover, TOCNF membranes, which are derived from sustainable resources, offer low cost and high separation efficiency, making them promising candidates to be used in the field of dye/salt wastewater treatment.

## Data Availability

Data are contained within the article.
